# Lead: Painting an Epidemic

**Published:** 2006-12

**Authors:** Ron Chepesiuk

The control of lead-based paint in residences has posed an ongoing public health challenge for decades. Now researchers have shown that several Asian countries are still selling paint with lead levels exceeding U.S. standards. The team warns in the September 2006 issue of *Environmental Research* that a worldwide ban on lead-based paint is needed to avoid perpetuating a global health epidemic. “By now, it is widely accepted around the world that lead should not be in paint,” says study head Scott Clark, a professor of environmental health at the University of Cincinnati College of Medicine.

Lead expert Bruce Lanphear, a professor of environmental health at Cincinnati Children’s Hospital Medical Center, says, “We know that many countries still do not have regulations for the lead content of either new paint or paint in existing housing. [Further,] it is both costly and difficult to reduce exposure from lead-based paint after it has been applied.” The United States has set limits of 600 ppm for lead in new paint for residential use and less than 5,000 ppm for paint in existing housing; paint containing at least 5,000 ppm lead is considered to be “lead-based.”

The effects of lead exposure depend on age (with children under 6 particularly vulnerable) and on whether the exposure is chronic or acute. In children, lead exposure can cause hyperactivity, anemia, brain damage, and mental retardation, while adults may suffer increased blood pressure, hearing and vision impairment, and nerve damage.

Clark and colleagues analyzed 80 consumer paint samples of various colors and brands from stores in India, China, Malaysia, and Singapore to compare their lead content to U.S. standards. They applied a single layer of each paint to a wooden block, then transported the samples to the University of Cincinnati for analysis.

The percentages of paint samples exceeding the U.S. lead limit for new paint were 100%, 72%, 56%, and 9% for India, Malaysia, China, and Singapore, respectively. The first three countries have no regulatory limits in place, whereas Singapore limits lead in new paint to 600 ppm. Clark says some multinational paint companies offered leaded paints in the countries without lead regulations at the same time that they sold lower-lead paint in Singapore. Noting that many U.S.-based paint manufacturers sell their products in Asia and have partners in the region, he says, “American companies need to promote U.S. environmental standards abroad and encourage their partners to lower the lead content in paint and other consumer products.”

Stephen R. Sides, vice president for environmental health and international affairs with the Washington, DC–based National Paint and Coatings Association, says, “We are sharing the findings of Dr. Clark’s study with the U.S. paint industry to make it aware of the lead paint–related concerns and public health issues in Asia. Hopefully, U.S. paint companies will ensure that their products aren’t contributing to the problems.”

